# Common variants of the beta and gamma subunits of the epithelial sodium channel and their relation to plasma renin and aldosterone levels in essential hypertension

**DOI:** 10.1186/1471-2350-6-4

**Published:** 2005-01-20

**Authors:** Tuula Hannila-Handelberg, Kimmo Kontula, Ilkka Tikkanen, Tuula Tikkanen, Frej Fyhrquist, Karri Helin, Heidi Fodstad, Kirsi Piippo, Helena E Miettinen, Jarmo Virtamo, Tom Krusius, Seppo Sarna, Ivan Gautschi, Laurent Schild, Timo P Hiltunen

**Affiliations:** 1 Department of Medicine, University of Helsinki, and Biomedicum Helsinki, University of Helsinki, 00290 Helsinki, Finland; 2 Department of Epidemiology and Health Promotion, National Public Health Institute, 00300 Helsinki, Finland; 3 The Finnish Red Cross Blood Service, 00310 Helsinki, Finland; 4 Department of Public Health, University of Helsinki, 00014 Helsinki, Finland; 5 Institute of Pharmacology and Toxicology, University of Lausanne, 1005 Lausanne, Switzerland; 6 Helsinki University Central Hospital, Jorvi Hospital, 02740 Espoo, Finland

## Abstract

**Background:**

Rare mutations of the epithelial sodium channel (ENaC) result in the monogenic hypertension form of Liddle's syndrome. We decided to screen for common variants in the ENaC βand γ subunits in patients with essential hypertension and to relate their occurrence to the activity of circulating renin-angiotensin-aldosterone system.

**Methods:**

Initially, DNA samples from 27 patients with low renin/low aldosterone hypertension were examined. The DNA variants were subsequently screened for in 347 patients with treatment-resistant hypertension, 175 male subjects with documented long-lasting normotension and 301 healthy Plasma renin and aldosterone levels were measured under baseline conditions and during postural and captopril challenge tests.

**Results:**

Two commonly occurring βENaC variants (G589S and a novel intronic i12-17CT substitution) and one novel γENaC variant (V546I) were detected. One of these variants occurred in a heterozygous form in 32 patients, a prevalence (9.2%) significantly higher than that in normotensive males (2.9%, p = 0.007) and blood donors (3.0%, p = 0.001). βENaC i12-17CT was significantly more prevalent in the hypertension group than in the two control groups combined (4.6% vs. 1.1%, p = 0.001). When expressed in *Xenopus *oocytes, neither of the two ENaC amino acid-changing variants showed a significant difference in activity compared with ENaC wild-type. No direct evidence for a mRNA splicing defect could be obtained for the βENaC intronic variant. The ratio of daily urinary potassium excretion to upright and mean (of supine and upright values) plasma renin activity was higher in variant allele carriers than in non-carriers (p = 0.034 and p = 0.048).

**Conclusions:**

At least 9% of Finnish patients with hypertension admitted to a specialized center carry genetic variants of β and γENaC, a three times higher prevalence than in the normotensive individuals or in random healthy controls. Patients with the variant alleles showed an increased urinary potassium excretion rate in relation to their renin levels.

## Background

Epidemiological studies have shown a significant correlation of blood pressure levels in close relatives and higher concordance values for occurrence of hypertension in monozygotic vs. dizygotic twins, and thus support the idea that genetic factors influence susceptibility to essential hypertension [1]. While recent molecular genetic studies have provided compelling evidence for mutations in at least seven different genes underlying rare forms of monogenic hypertension [1,2], progress in the understanding of the molecular basis of human essential hypertension has been much slower. Hundreds of case-control studies have suggested hypertension-related genetic variants of which only a few if any have tolerated replication analyses; it is possible that common variants of angiotensinogen [3], α-adducin [4] and the G-protein β subunit [5] confer susceptibility to elevated blood pressure in at least some populations. Since 1999, a number of genome-wide linkage studies in families with multiple affected hypertensive members have been published with highly varying results (for review, see [6]). Recent large-scale searches for genes predisposing to hypertension, published as a recent series of articles [7-11], failed to identify definite linkage of hypertension to any chromosomal locus, although some DNA regions showing suggestive linkage were disclosed. Reasons for these disappointing data were put on the account of the unsuitability of using a single-locus linkage strategy for a multifactorial genetic disease, inherent genetic heterogeneity of essential hypertension, and complex interplay of genetic and environmental factors underlying regulation of blood pressure variation [12].

Disappointments in the previous strategies justify alternative approaches in which a better phenotyping of the study individuals is connected to their targeted molecular genetic characterization. There are several features that collectively make the genes encoding the beta (βENaC) and gamma (γENaC) subunits of the kidney tubular epithelial sodium channel as serious candidates for susceptibility genes of low-renin human essential hypertension. First, gain of function mutations in β and γ ENaC subunits cause Liddle's syndrome, a well-known monogenic form of human hypertension associated with low renin activity and low plasma aldosterone level [13-15]. Second, common βENaC variants occur in increased frequency in hypertensive black individuals [16-18]. Third, an extensive locus-targeted study on hypertensive family members demonstrated a significant linkage of hypertension to chromosome 16q region harboring both the βENaC and γENaC genes [19]. These data prompted us to carry out a search for common variants of these two genes in Finnish hypertensive patients who were admitted to a special center because of treatment-resistant hypertension and whose renin-aldosterone system was systematically examined. These circumstances provided a group of hypertensive patients, in which secondary forms of hypertension were effectively excluded and who originated from a genetic isolate. Our data suggest that common variants of the ENaC subunits confer susceptibility to human essential hypertension.

## Methods

### Patients with hypertension

**Figure 1:**
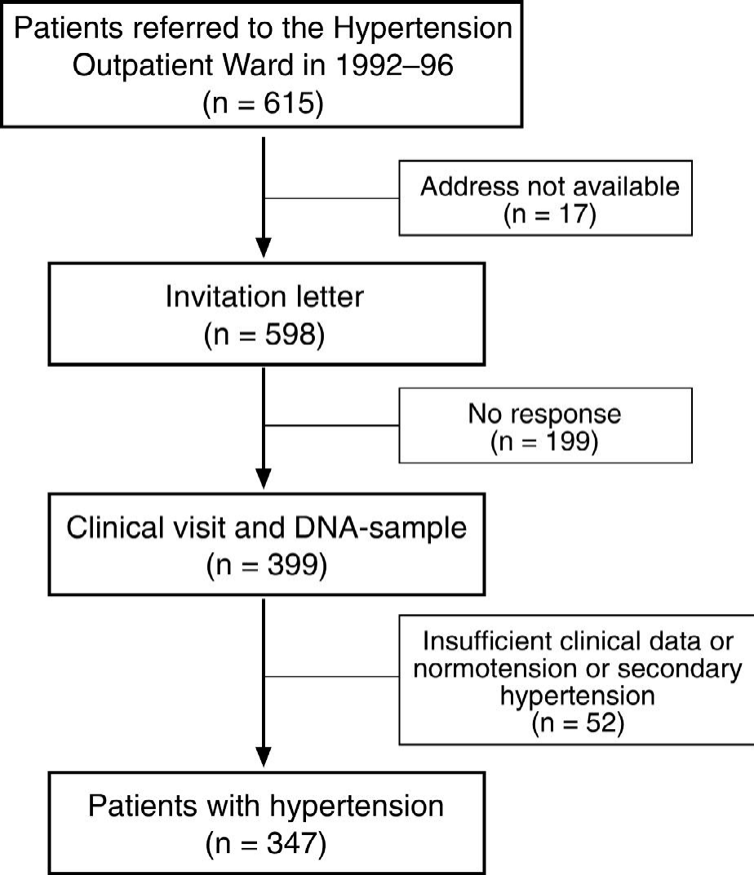
Flow diagram of the recruitment of the patients with primary hypertension.

The clinical records of all consecutive patients with hypertension (n = 615) referred to the Hypertension Outpatient Ward, Helsinki University Central Hospital, between 1992–96 were reviewed. Moderate-to-severe hypertension, suspicion of secondary forms of hypertension, or hypertension resistant to drug treatment were causes to the admittance. A letter with request to donate a blood sample for genetic studies on hypertension was sent to those 598 individuals whose address became available in 1998. A total of 399 individuals (67%) of these responded and were subsequently examined at the Hypertension Outpatient Ward in 1998 to 1999. Clinical and family histories were recorded, and venous blood samples taken for DNA analysis. Based on the previous documents and current examinations, altogether 52 individuals were excluded from the present study: clinical records were missing or insufficient in four cases, 22 subjects were considered as normotensive, while 26 were judged to have a secondary form of hypertension. The latter group consisted of the following cases: renal artery stenosis (n = 12), adrenal cortical adenoma (n = 3), hydronephrosis (n = 2), pheochromocytoma (n = 1), IgA glomerulonephritis (n = 1), non-specific chronic glomerulonephritis (n = 1), LED nephritis (n = 1), diabetic nephropathia (n = 1), chronic pyelonephritis (n = 1), hypernephroma (n = 1), fibromuscular dysplasia (n = 1), unspecified renal failure (n = 1). The remaining 347 patients (186 females and 161 males, mean age 49.3 years, SD ± 10.0) comprised our final cohort of patients with moderate-to-severe essential hypertension. Antihypertensive drug treatment was in use in 283 (82%) of the patients (diuretics, 19%; beta-blocking agents, 35%; calcium-channel blockers, 21%; ACE-inhibitors, 33%; angiotensin receptor antagonists, 1%). At least two concomitant drugs were used by 24% of the patients. A flow-chart of the study design is illustrated in Fig. 1.

### Control individuals

#### Blood donors

DNA was extracted from 301 randomly selected healthy blood donors aged 40–50 years (mean, 45 years) visiting the Finnish Red Cross Blood Transfusion Service. Their residences represent the same capital area from which the hypertensive patients originated.

#### Normotensive controls

These individuals were selected from the participants in the Alpha-Tocopherol, Beta Carotene (ATBC) study [20] using the criteria described previously [21]. In brief, a total of 27271 male smokers (aged 50 to 69 years) with no previous history of myocardial infarction were initially recruited for a cancer prevention trial. DNA samples were available from 70% of the original participants. We picked up all the available blood samples from those fulfilling the following criteria: no known hypertensive disorder, no antihypertensive drugs ever in use, systolic and diastolic blood pressure values ≤ 128 and ≤ 84 mmHg, respectively, at each blood pressure measurement, repeated five times at one-year intervals during a five-year follow-up. We ended up with 175 normotensive men whose mean systolic and diastolic blood pressures were 114.9 (SD ± 5.4) and 73.7 (SD ± 4.3) mmHg, respectively, during this five-year follow-up.

The Ethics Review Committee of the Helsinki University Central Hospital approved this study, and all patients and controls gave their informed consent.

### Laboratory measurements in the hypertensive patients

The patients were advised to stop using estrogens and spironolactone at least 4 weeks before the tests, diuretics and prostaglandin inhibitors at least two weeks before the tests, and β-adrenergic antagonists and ACE inhibitors at least one week before the test. The only antihypertensive agents permitted at the time of the test were calcium channel blockers. Some of the patients were on oral potassium supplementation because of hypokalemia. The mean baseline blood pressure level at the time of captopril test was 139 ± 16/94 ± 10 mmHg in those without any drugs (n = 79), and 142 ± 16/95 ± 11 mmHg in those with calcium channel blockers (n = 234).

Fasting blood samples were taken for determination of serum creatinine, uric acid, cholesterol, potassium, sodium and blood glucose concentrations. Urine samples for determination of the daily (24 h) excretion of potassium and sodium were collected. Most hypertensive patients (n = 298) underwent a test for the responsiveness of serum aldosterone level and plasma renin activity to postural change. To this end, the first blood sample was taken after at least 60 minutes of rest in supine position. After 2 hours of standing and moderate walking, a second blood sample was taken. This test was carried out at the inpatient ward in 220 cases and at the outpatient ward in 78 cases. Urinary electrolyte excretion rates were analyzed in 262 patients (26 ENaC variant carriers and 236 non-carriers) who did not use potassium supplementation.

One to three days later, a captopril challenge test (CCT) was carried out as described earlier [22]. This test was carried out in a total of 313 patients, and was performed at the inpatient ward in 229 cases and at the outpatient ward in 84 cases. CCT was started by sitting for at least 30 minutes, followed by oral administration of 50 mg captopril. Blood pressure in the non-dominant arm was measured at 15-minute intervals. Blood samples for the determination of plasma renin activity and serum aldosterone concentration were drawn immediately before and 60 minutes after captopril administration.

### DNA analysis

Genomic DNA was extracted from peripheral venous blood using standard techniques. For targeted search for ENaC variants postulated to be associated with increased channel activity, we chose to sequence the exons 13 coding for the carboxyterminal domains of βENaC (amino acids 515–640) and γENaC (amino acids 524–649), as well as the 5'-flanking intronic regions, using oligonucleotide primers, PCR (polymerase chain reaction) conditions and sequencing instruments described previously by us [21]. DNA samples of 27 patients of those 399 initially visiting the Hypertension Outpatient Ward showing the lowest plasma renin activities (median 0.7 μg/L/h at 0 minutes and 0.9 at 60 minutes) and serum aldosterone concentrations (median 236 pmol/L at 0 minutes and 212 at 60 minutes) during CCT were selected for this initial step.

Specific PCR-based methods were set up for assaying the three ENaC variants detected during the present study. After PCR of the βENaC fragment, the βENaC -i12 -17CT and βENaC G589S variants could be assayed simultaneously. An aliquot (8 μl) of the PCR product was digested with 3.0 U of *Alu*I (New England Biolabs, Beverly, Massachusetts, USA), followed by analysis of the cleavage products on a 12% polyacrylamide gel. The wild-type (wt) allele results in longest fragments of 266 and 137 bp, while the variant allele produces fragments of 266 and 147 bp for βENaC -i12 -17CT, and 240 and 137 bp for βENaC G589S. For the γENaC V546I variant, 2.0 U of *Sfa*NI (New England Biolabs) was used and the cleavage products were analyzed on a 2% agarose gel. The resulting fragment sizes were 357 and 77 bp for the wild-type allele and 279, 78 and 77 bp for the variant allele.

For studies on the possible splicing errors brought about by the βENaC i12-17 variant, we collected lymphocytes from two subjects heterozygous for this variant and one control subject. Total lymphocytic RNA was isolated using Qiagen RNeasy kit (Qiagen, Valencia, California, USA), and first strand synthesis was performed using Superscript system for RT-PCR (Invitrogen Corporation, Carlsbad, California, USA). For gene-specific PCR, we used two sets of primers amplifying a fragment extending from exon 12, either to exon 13 (180 bp) or 141 bp downstream of exon 13 (551 bp). The amplified products were run on a 12 % polyacrylamide gel and visualized by ethidium bromide. The amplified fragments were also sequenced to exclude presence of any splicing defects. Additionally, possible splicing differences between the wild-type and i12-17 variant of βENaC were studied *in silico *using GrailEXP v3.3 (Perceval) exon prediction program [23].

### Site-directed mutagenesis and functional characterization of the ENaC variants

The human βENaC cDNA and γENaC cDNA cloned into the pBSK-SP6-globin vector were used in construction of the βENaC G589S and γENaC V546I mutations, respectively. Site-directed mutagenesis was performed using the Transformer site-directed mutagenesis kit (Clontech Laboratories, East Meadow Circle, California, USA). Mutagenic primers used were 5'-cacaccaacttt**A**gcttccagcctg-3' and 5'-gctgctctgttgtctgc**A**tcatcgagatcatcgagg-3' for the G589S and V546I mutations, respectively. The primer 5'-ccctcgctcg**T**gtgatctggt-3', which mutates the *Xho*I restriction enzyme site in the pBSK-SP6-globin vector, was used as the selection primer in the mutagenesis reactions. The mutagenic clones were sequenced to confirm the presence of the mutations and to exclude undesired errors during mutagenesis.

Healthy stage V and VI *Xenopus *oocytes were injected with mRNAs encoding the β human (h)ENaC wild-type or βG589S hENaC mutant, the γhENaC wt or γV546I hENaC mutant together with the mRNA encoding the αhENaC wt. The total amount of mRNA encoding the three αβγ ENaC subunits was 10 ng. Electrophysiological measurements were taken at 16–24 hours after injection. ENaC activity was assessed by measurement of the amiloride-sensitive current (I_Na _in μA) recorded at -100 mV with a two-electrode voltage clamp amplifier (TEV-200, Dagan Corp.) in a standard solution containing 110 mmol/L NaCl, 1.8 mmol/L CaCl_2_, 10 mmol/L HEPES-NaOH, pH 7.35. The amiloride concentration used was 5 μmol/l in the bath solution. Four batches of oocytes were obtained from different *Xenopus *frogs in which 5 to 7 oocytes were tested for each αβγ ENaC wt and ENaC variants.

### Statistical analysis

The renin and aldosterone values were nonnormally distributed, as analyzed using skewness, kurtosis and Kolmogorov-Smirnov tests. Therefore, nonparametric tests (Mann-Whitney's U) were used in the statistical analyses, and interquartile (IQ) range and median are used to describe the distributions of target variables. When covariates were included in the analyses, ANCOVA with ranks or logarithm-transformed values of the variables was used. Chi square test, or Fisher's exact test if observed frequency in any cell was less than five, were used for the frequency analysis of the variants. Logistic regression was used to obtain age and gender adjusted odds ratios for hypertension in ENaC variant carriers vs. non-carriers. All data was analyzed using statistical SPSS program (version 11.0). Because of relatively small variant group sizes, the primary analyses were performed with all variant groups combined. Secondarily, the variant groups were also compared separately with the wild-type ENaC group.

## Results

### Identification of three common ENaC variants and screening for their presence in the three different study groups

**Figure 2:**
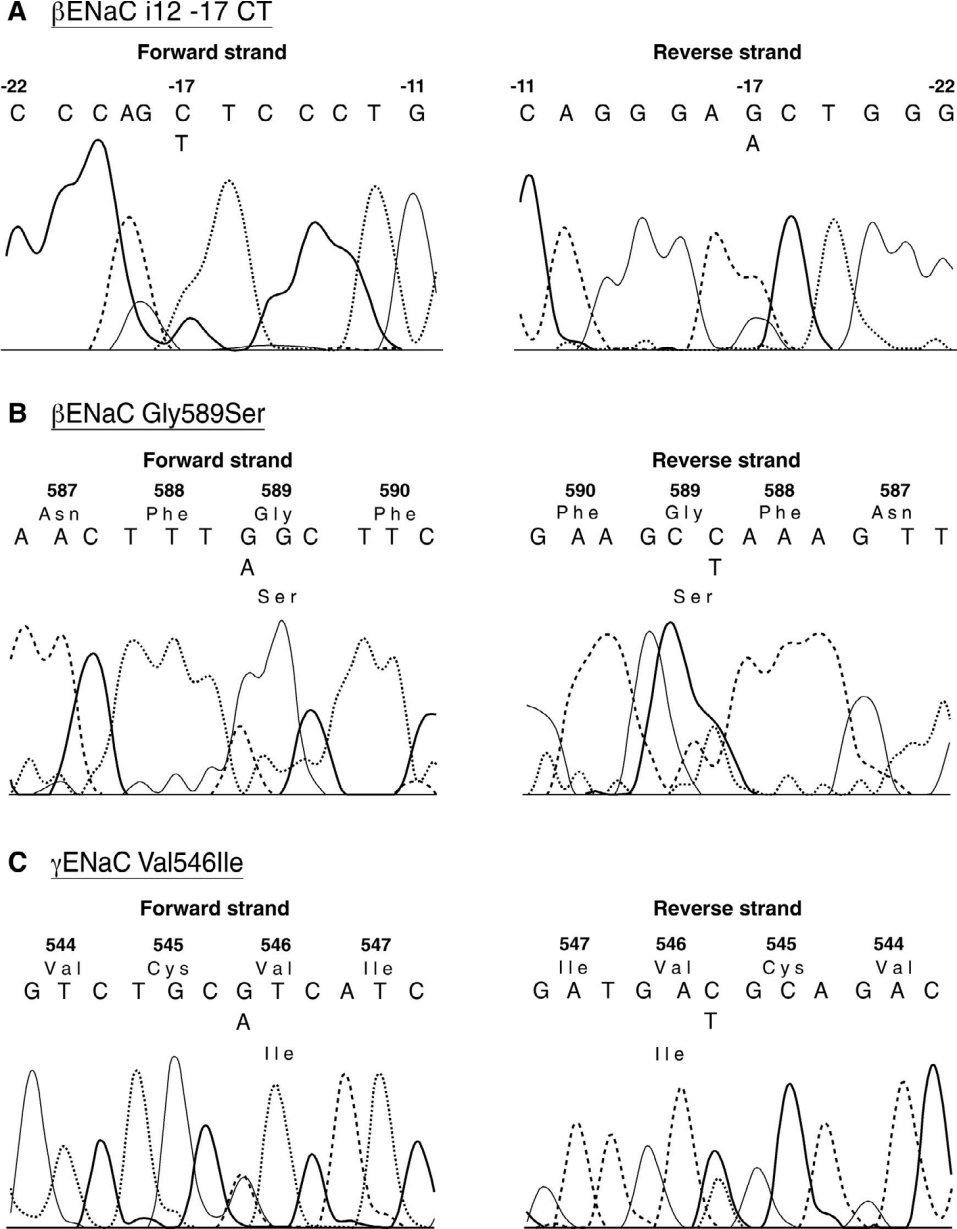
**Sequence analysis of the variant β and γENaC alleles. **Chromatograms from both sequencing directions, nucleotide substitutions and predicted amino acid changes from three different hypertensive patients are shown.

DNA samples of 27 hypertensive patients with lowest renin activities and aldosterone concentrations were initially selected for targeted search for ENaC variants. The sequencing strategy chosen permits detection of mutations and polymorphisms in the entire coding parts of exons 13 of β and γENaC genes, as well as 26 or 43 nucleotides at the 3'-ends of introns 12. Three different single-nucleotide substitutions were detected, two in the βENaC and one in the γENaC subunit (Fig. 2). Four out of the 27 samples showed a previously unreported substitution of T for C in intron 12 of the βENaC gene (i12-17CT), located 17 nucleotides upstream of the 5'-end of exon 13. In one DNA sample a single G to A substitution changed the codon 589 of βENaC from GGC to AGC, predicted to result in a substitution of serine for glycine (G589S). This variant has been described previously [24,25]. Upon screening of exon 13 of the γENaC gene for mutations, one sample was detected with a novel point mutation changing codon 546 from GTC to ATC, which results in a substitution of isoleucine for valine (V546I).

We next conducted a search for these three ENaC variants in our whole material of patients with essential hypertension (n = 347), normotensive males (n = 175) and randomly chosen blood donors (n = 301) (Table 1). Altogether, we identified 46 heterozygous carriers of these variant alleles, but no homozygous or compound heterozygous individuals. Their prevalence was significantly different in the three study groups (χ^2 ^= 15.0, p = 0.0006). Subanalysis of the three groups indicated that the variant allele frequency was higher among the hypertensive patients (9.2%) than in normotensive males (2.9%; p = 0.007) or blood donors (3.0%; p = 0.001), while in the latter two groups it was similar (Table 1). When frequencies of the individual gene variants in the hypertensive patients were compared to those in the two other groups (normotensive males and blood donors) combined, the βENaC i12-17CT variant was found to occur significantly more often among the hypertensive patients than in other groups (p = 0.001) whereas the differences in the prevalences of βENaC G589S (p = 0.15) and γENaC V546I (p = 0.14) did not reach statistical significance.

### Clinical characteristics of the variant allele carriers and non-carriers

Clinical and laboratory data of the hypertensive patients grouped according to their carrier status of the three ENaC variants detected are summarized in Table 2. There were no significant differences, associated with carrying a variant allele, in the sex, age or BMI of the hypertensive patients, nor their serum creatinine, lipid, potassium or sodium levels. Variant alleles did not seem to associate with cerebrovascular events or diabetes among the hypertensive patients, but small numbers prevent definitive conclusions (Table 2). Our original study protocol was not designed to disclose health information of the two reference groups (normotensive males and healthy blood donors), and a similar comparison of variant allele carriers and non-carriers in these groups is therefore not feasible.

### Relation of the variant ENaC alleles to the activity of the renin-aldosterone system

**Figure 3:**
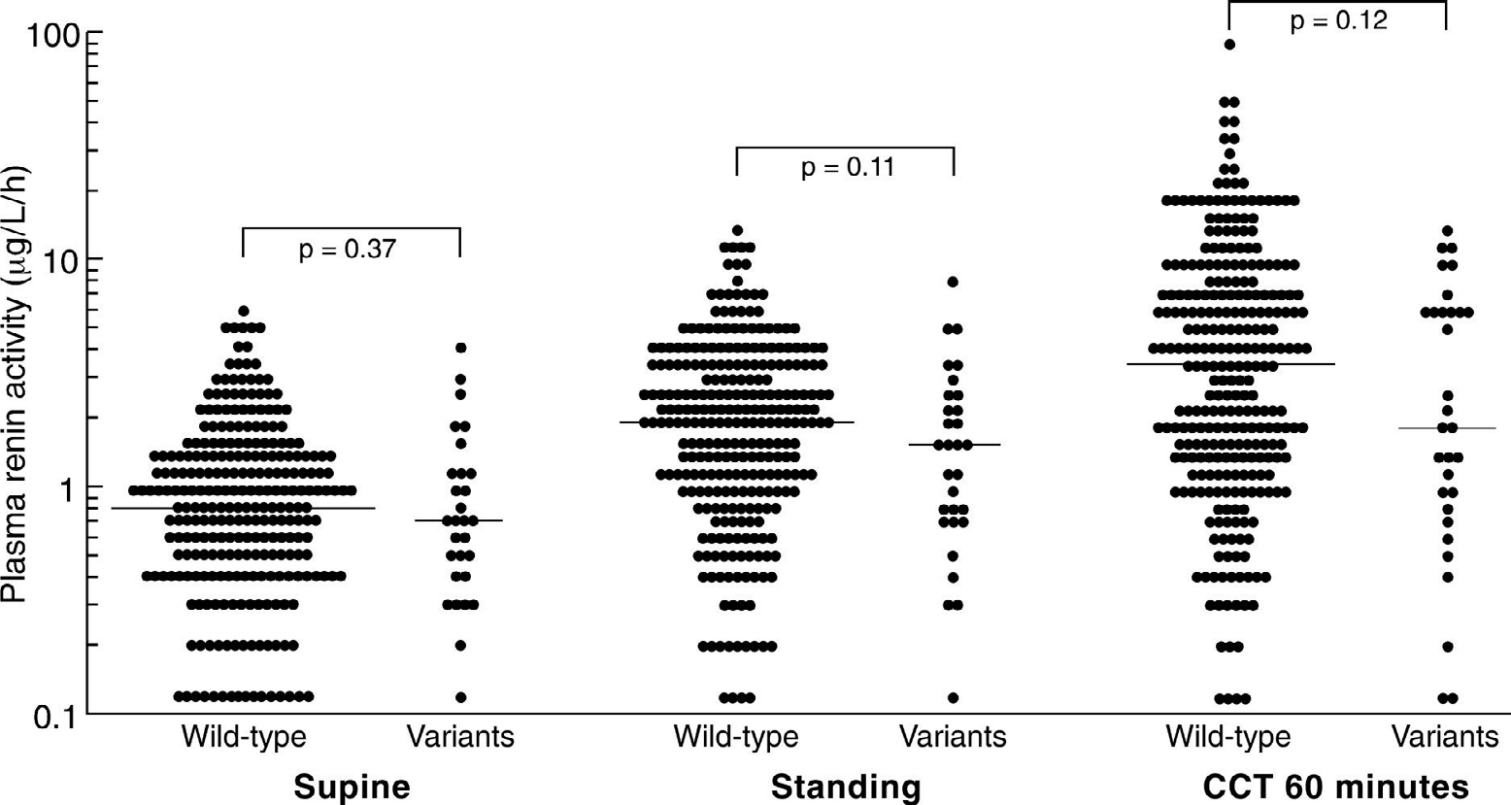
**Renin values in postural test and after captopril administration. **Individual plasma renin activities at supine, upright, and in response to captopril administration (CCT; 60-minute values) in carriers and non-carriers of the three ENaC variant alleles. The horizontal bars indicate the median renin values in each group.

The dynamics of the circulating renin and aldosterone levels in most hypertensive individuals were studied during two challenge tests: during a supine-upright postural test and in response to captopril administration. Baseline plasma renin activity was very similar in the patients with and without variant alleles, whether investigated during the postural test or captopril administration (Table 3). Plasma renin levels after attainment of upright posture (p = 0.11) and captopril administration (p = 0.12) were not significantly different among carriers and non-carriers of the ENaC variants (Table 3, Fig. 3). Plasma aldosterone levels did not significantly vary according to the ENaC variant carrier status (Table 3).

**Figure 4:**
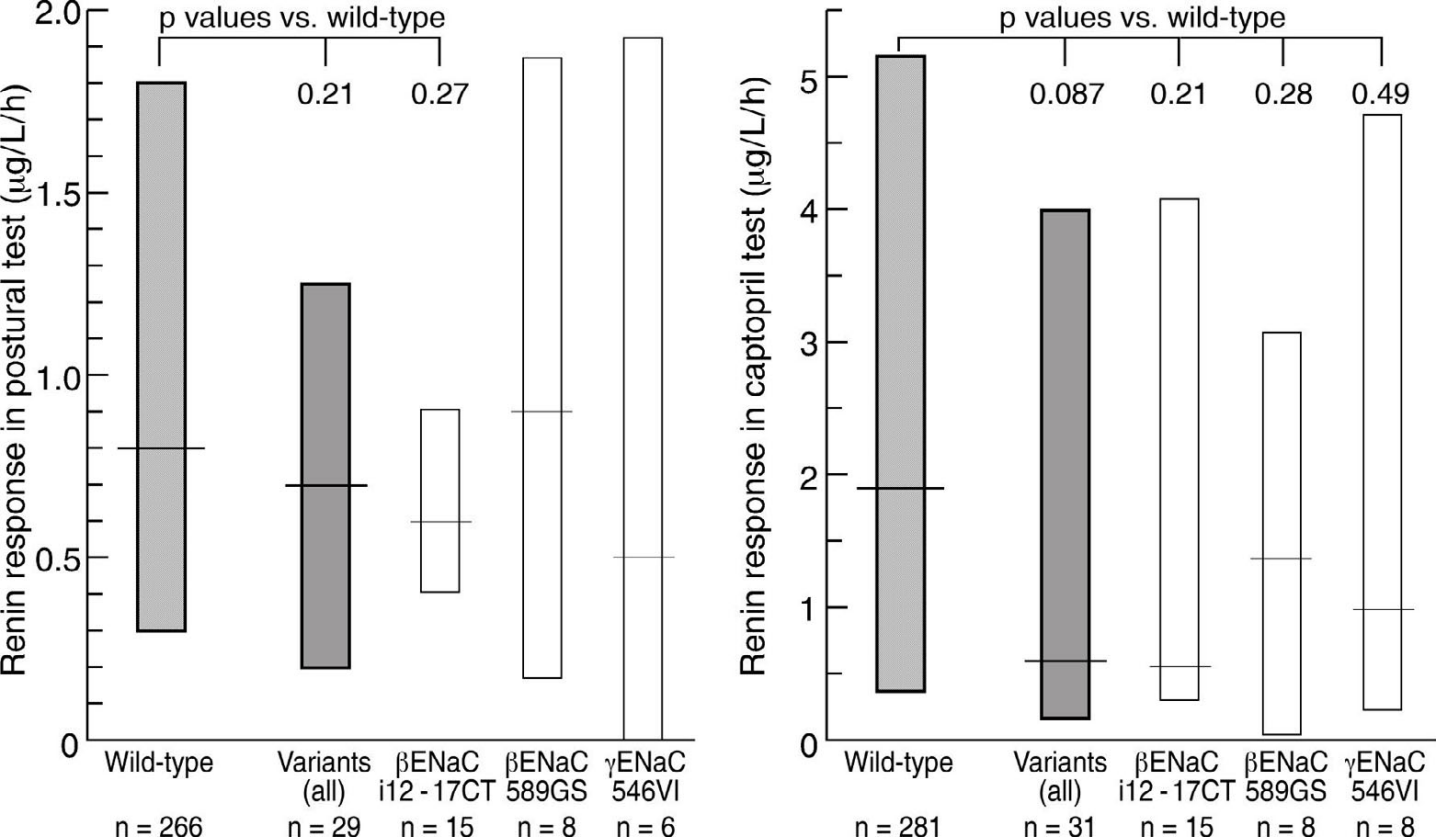
**Renin responses in postural and captopril tests. **Plasma renin responses (median and interquartile ranges) during the postural and captopril challenge tests (stimulated values minus baseline values in both cases) in carriers and non-carriers of the variant ENaC alleles.

We also analyzed renin responses (stimulated value minus baseline value) in the two tests according to the ENaC variant carrier status. We found some evidence of a blunted renin response to both postural (p = 0.21) and captopril (p = 0.087) challenge tests in carriers of variant alleles compared to non-carriers, but there was wide interindividual variation in the test results (Fig. 4). Use of covariates (urinary sodium excretion, age and BMI) did not cause significant changes in the results of these analyses.

We next related the activity of circulating renin-aldosterone system to sodium-potassium homeostasis in ENaC variant carriers and non-carriers. Serum sodium and potassium concentrations in these two groups of hypertensive patients were similar (Table 2). Urinary sodium excretion rate was not associated with the ENaC polymorphisms studied (data not shown). Urinary potassium excretion rates were not statistically significantly different in patients with (median, 83 mmol/day) and without (median, 79 mmol/day, p = 0.23) ENaC variants (Table 4). However, when daily potassium excretion (dU-K, in mmol/day) was related to plasma renin activity (in μg/L/h), as mirrored by the renin levels during the postural challenge test, a significant difference was noticed: the median dU-K/renin ratios in the variant carriers vs. non-carriers were 114 vs. 92 when supine (p = 0.29) and 56 vs. 38 when upright (p = 0.034) (Table 4); the corresponding values for the average (mean of supine and upright) dU-K/renin ratios were 74 and 51, respectively (p = 0.048) (Table 4). A similar analysis of dU-K/plasma aldosterone ratios demonstrated higher ratios in female variant carriers vs. non-carriers for supine (p = 0.16), upright (p = 0.014) and the average values (p = 0.012), while no significant differences were seen in males. Collectively, these data suggests that hypertensive individuals carrying the ENaC variants tend to excrete increased amounts of potassium in relation to prevailing plasma renin and aldosterone levels.

### Molecular characterization of the ENaC variants

We tested whether the βG589S or γV546I have any functional impact on ENaC expressed in *Xenopus *oocytes, the most commonly used expression system for ENaC functional studies. When αβγ ENaC subunits were co-expressed to obtain maximal channel activity, neither the βG589S nor γV546I affected ENaC activity as measured by the amiloride-sensitive Na^+ ^currents. In other words, these data indicate that the current carried by Na^+ ^ions through ENaC channels present at the cell surface is similar for ENaC wild-type and mutant channels, indicating that the βG589S and γV546I mutations have no detectable functional consequences on ENaC activity, at least when expressed in *Xenopus *oocytes.

In order to clarify whether the C-T substitution at position -17 of the intron 12 of the βENaC could affect mRNA splicing, cDNA was synthesized from an RNA fraction prepared from lymphocytes of two hypertensive patients heterozygous for the βENaC i12-17CT mutation and a control individual without this gene variant. Primer pairs for reverse transcription were designed in a way permitting identification of a possible failure to splice intron 12 properly. Regardless of the primer pairs used, similar DNA fragments were generated from the samples of the βENaC i12-17CT carriers and control subject (data not shown). Furthermore, sequence analysis of the amplified DNA fragments revealed the presence of only normally spliced DNA sequence in the βENaC i12-17CT carriers. Furthermore, *in silico *analysis of the βENaC wild-type and variant DNA sequences suggested no differences in exon splicing. However, this analysis is only of predictive value and does not exclude a splicing defect introduced by the variant nucleotide in renal tissue.

## Discussion

The present study indicates that three common variants of the kidney epithelial sodium channel ENaC occur approximately three times more often in patients with moderate-to-severe essential hypertension compared to normotensive males and While direct *in vitro *studies have failed to demonstrate a gain-of-function for these ENaC variants, their association with an increased urinary potassium loss in relation to existing plasma renin activity suggests that in the long run *in vivo *they may result in sodium retention, suppression of renin and aldosterone levels and hypertension.

A large number of common and rare polymorphisms of the α-, β-and γENaC have been described in different populations (reviewed in [26] and [27]), but their pathophysiologic role, if any, has remained obscure, at least in the White populations. A systematic search in approximately 500 hypertensive probands, mostly of Caucasian origin, disclosed seven variants of the βENaC and six variants of the γENaC subunit, but no variant, with the possible exception of the βENaC G589S substitution, showed an increased ENaC activity *in vitro*, nor showed cosegregation with hypertension [24,28]. The G589S variant was also identified in a Swedish hypertensive patient [25].

**Figure 5:**
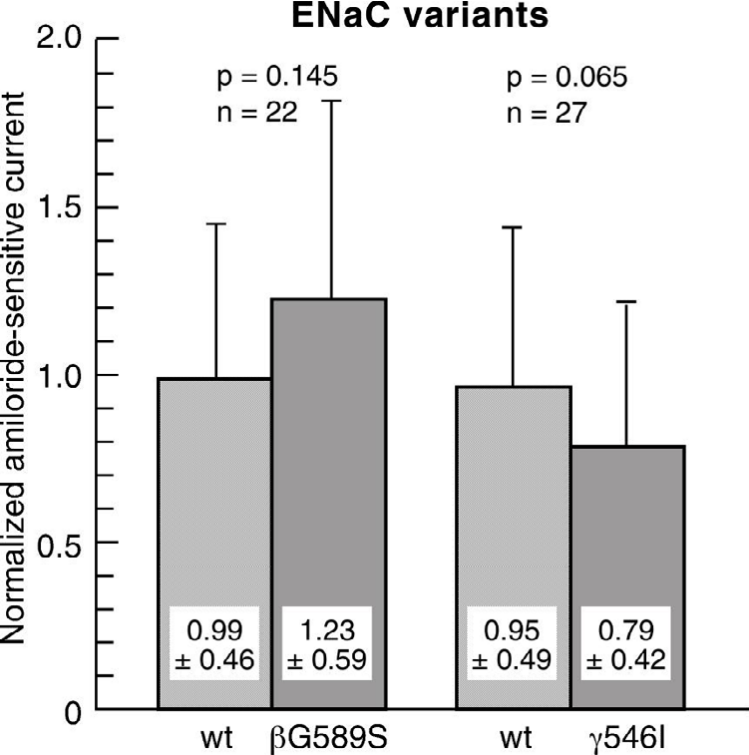
**Channel activity of βENaC G589S and γENaC V546I variants in vitro. **Comparison of channel activity of hENaC wild-type, and the βhENaC G589S and γhENaC V546I variants, when expressed in *Xenopus *oocytes. ENaC activity was measured as amiloride-sensitive Na^+ ^current. Absolute currents were 5.09 ± 0.98 and 6.75 ± 1.23 μA for ENaC wt in the two series of experiments (number of oocytes given in parentheses).

Two amino acid variants, βENaC G589S and γENaC V546I, both occurred with a frequency of about 2% in the hypertensive patients but in only 1% of the background population or normotensive males. The G589 is located in the poorly conserved cytoplasmic carboxyterminal portion of βENaC, 27 amino acids upstream of the functionally important PY motif. Persu et al. [24] identified the same substitution in a hypertensive female with mild hypokalemia and suppressed plasma renin activity. Using measurements of sodium channel activity and amiloride-sensitive sodium flux in *Xenopus *oocytes, these investigators were able to show a borderline 1.3 to 1.5-fold increase in activity for the G589S variant compared with the wild-type subunit. A similar trend was noticed in our experiments (Fig. 5). It remains possible that the functional expression of ENaC in *Xenopus *oocytes is not sensitive enough to detect subtle increases in ENaC activity, as it could well be the case for βG589S ENaC variant, and only mutations leading to large changes in ENaC activity are liable to be detected. On the other hand, even minute changes in ENaC may result in significant *in vivo *effects when operating for decades under the influence of unfavorable living habits or variants of other modifier genes promoting salt reabsorption. Accordingly, the βENaC G589S could confer some susceptibility to low-renin hypertension, but more data on untreated patients and families are needed.

The γENaC V546I substitution is located in the second transmembrane domain of the ENaC subunit, and it has not been described previously. Seven out of the eight carriers were females, and their renin and aldosterone levels were very similar to those in non-carriers. When expressed *in vitro *in *Xenopus *oocytes, this substitution did not result in an increase in sodium current (Fig. 5). It is not possible at present to deduce whether the V546I variant constitutes a pathophysiologically significant allele by itself or merely a genetic marker conferring susceptibility to hypertension.

The C→T variant of the nucleotide -17 of intron 12 of βENaC is a novel one and, interestingly, it was present in 4.6% of the hypertensive patients but in only 1% of the 301 random blood donors (p = 0.009) and 175 normotensive males (p = 0.043). Patients with this variant allele displayed the lowest plasma renin levels and responses of all the subgroups examined (Table 3, Fig. 4), but due to large interindividual variation the differences were not statistically significant. This βENaC variant may have remained undetected in earlier studies as they have mostly employed 5'-PCR primers annealing at the region containing this substitution. Theoretically, a mutation at this site of an intron could affect RNA splicing. We explored this possibility by reverse transcription-PCR experiments of RNA samples from two variant carriers and a control individual, prepared from peripheral lymphocytes known to express βENaC [29]. We could not demonstrate a splicing error, but since homozygous individuals were not available for studies, we may have missed subtle changes. Furthermore, it is not known how well βENaC mRNA splicing in lymphocytes reflects the mechanism in kidney epithelial cells. Another possibility is that the DNA region around the variant nucleotide -17 of intron 12 contains interaction site for regulatory factors affecting transcription of βENaC in tubular cells, or the i12-17CT variant may be in linkage disequilibrium with some yet unidentified mutation present elsewhere in the βENaC or in the closely linked γENaC gene.

The fact that we did not find hypokalemia or statistically significant suppression of renin levels in our patients with variant ENaC alleles does not abandon the hypothesis that they act as subtle genes conferring liability to sodium retention and hypertension during lifetime. In fact, even in cases with unequivocal Liddle's syndrome due to activating ENaC mutations the penetrance of disease phenotype is variable, with inconstant occurrence of hypertension, hypokalemia and suppressed renin levels from patient to patient [13,30-32]. This suggests that Liddle's syndrome may represent an intermediate between single-gene and complex genetic diseases, necessitating the effect of extrinsic factors, such as substantial salt intake or other modifier genes, to complete the spectrum of syndrome manifestations. It is of particular note that molecular variants resulting in increased ENaC activity may occur outside the cytoplasmic PY motif that long was considered as a critical domain to be affected in Liddle patients [21,33,34].

Our present data are supported by findings of Rayner et al. [18] who recently discovered another βENaC variant (R563Q), which is located in the cytoplasmic domain just adjacent of the cell membrane and was found to be strongly associated with low-renin, low-aldosterone hypertension in a South African black population. Unfortunately, functional characterization of the R563Q variant was not carried out. Previously, another βENaC variant (T594M) was identified in the African Americans [35]. Although initially not linked to elevated blood pressure in the Blacks [35], subsequent studies in a London black population suggested a positive association with hypertension [16,36]. The T594M substitution was reported to result in an increased responsiveness to a cAMP analog due to loss of protein kinase C inhibition of the ENaC [35,37], but other studies have failed to show increased sodium currents in transfected cells [24]. An additional βENaC variant (G442V) present almost exclusively in Blacks has also been suggested to be associated with biochemical alterations compatible with increased ENaC activity in vivo [17].

Our present results and the previous data summarized above suggest that subtle β and γENaC variants do exist in the population that may variably result in elevated ENaC activity, suppression of plasma renin and aldosterone levels, urinary loss of potassium, and elevated blood pressure levels. Individual patients may variably manifest either only one or several of these features, and in some of the variant carriers these parameters may be entirely normal. It will be of interest to test the effectiveness of amiloride in our patients with the βG589S, i12-17CT and γV546I variants as an antihypertensive drug as this specific ENaC antagonist was shown to control blood pressure as well as increase plasma renin, aldosterone and potassium levels in black hypertensive individuals carrying the βT594M allele [38].

There are certain limitations in our study. First, our hypertensive patients represent a highly selected type of patients, since they were recruited by admittance to a specific center focusing on problems in conventional treatment. The clinical study protocol was initially designed for studies on screening for renovascular hypertension in a population, which explains the use of captopril test in the test panel. Unfortunately, urinary aldosterone levels, integrating aldosterone secretion rate over a longer observation period and serving as a valuable marker of Liddle's syndrome [13,31], in particular when related to urinary potassium excretion levels [30],were not studied systematically. Our single-point plasma renin and aldosterone measurements may have been liable to incidental variations in their plasma levels, and prevent direct comparison to previous studies relying on urinary aldosterone assays. Second, our normotensive reference population comprised of male patients only. However, we had the advantage of picking up the extreme lowest end, as regards systolic and diastolic blood pressure levels in the absence of any antihypertensive drugs, from a very large material of more than 27000 individuals [20]. Third, due to ethical limitations of the study design, we did not have access to the clinical data of the subjects in the two reference groups (normotensive males and healthy blood donors); it would have been of interest to review the health data of ENaC variant carriers in these two groups. Fourth, our study had limited statistical power for several of the questions asked, particularly when either genetic variant was analyzed alone in carriers versus non-carriers. Therefore, for several of the questions asked in this study these variants may well have only modest effects, too small to be detected using the parameters of the present study. Fifth, pooling of the three genetic variants may represent an oversimplification, as it is uncertain whether these three variants exert similar effects on the various endpoints studied. Finally, our statistical analyses were not corrected for multiple comparisons and therefore some of the results observed in this study could represent chance findings rather than real phenomena. However, this is probably not the case for the observed increased frequency of ENaC genetic variants in hypertensive patients versus normotensive males and blood donors, because this was the primary hypothesis tested and the p values for these comparisons were 0.007 and 0.001, respectively.

## Conclusions

We have demonstrated that almost 9% of Finnish patients with hypertension admitted to a specialized center carry genetic variants of β and γ subunits of the kidney epithelial sodium channel ENaC, a percentage three times higher than that in the normotensive individuals or random healthy controls. Patients with the variant alleles tended to have suppressed renin levels and renin responsiveness to challenging stimuli, and they showed a significantly increased urinary potassium excretion in relation to their renin levels. It will be important to study whether carriers of ENaC variants respond favorably to ENaC blockers (amiloride and triamterene).

## Competing interests

The authors declare that they have no competing interests.

## Authors' contributions

TH-H collected the clinical material, and participated in the DNA analyses and drafting of the manuscript. KK and TPH designed the study and drafted the manuscript. IT, TT, FF, KH designed the clinical chemical and hormonal assays, and participated in collection of the patient material. HF, HEM and KP participated in DNA analyses and bioinformatics. JV and TK collected the control populations and designed their studies. SS consulted in the statistical analyses and assisted in data handling. IG and LS carried out the electrophysiological studies and participated in drafting of the manuscript. All authors read and approved the final manuscript.

## Pre-publication history

The pre-publication history for this paper can be accessed here:



## Figures and Tables

**Table 1:** β and γ ENaC variants identified among the three study groups

	Hypertension n (%)	Normotensive males n (%)	Blood donors n (%)	Adjusted OR (95% CI)^1^
				
**All variants**	32 (9.2)	5 (2.9)**	9 (3.0)**	3.1 (1.6–6.0)
βENaC i12-17CT	16 (4.6)	2 (1.1)*	3 (1.0)**	4.6 (1.6–13.0)
βENaC G589S	8 (2.3)	2 (1.1)	3 (1.0)	2.4 (0.77–7.7)
γENaC V546I	8 (2.3)	1 (0.6)	3 (1.0)	2.2 (0.63–7.5)
				
**Non-carriers**	315 (90.8)	170 (97.1)	292 (97.0)	

*P<0.05 and **P<0.01 vs. Hypertension group.

^1^OR for hypertension (versus combined control groups) in ENaC variant carriers vs. non-carriers, adjusted for age and gender.

**Table 2:** Demographic and clinical features of the hypertensive subjects, according to their ENaC variant status

	**Carriers of the ENaC variants**				**Non-carriers**
	βENaC i12-17CT (n = 16)	βENaC G589S (n = 8)	γENaC V546I (n = 8)	All (n = 32)	(n = 315)

					
Female/male (n)	9/7	4/4	7/1	20/12	166/149
Age (y)	49.4 ± 8.5	49.3 ± 5.8	50.5 ± 9.0	49.7 ± 7.8	49.3 ± 10.2
BMI (kg/m^2^)	28.0 ± 4.9	28.2 ± 4.8	27.9 ± 3.8	28.0 ± 4.5	27.4 ± 4.9
Serum creatinine (μmol/L)	88 ± 18.9	91 ± 12.8	85 ± 17.1	88 ± 6.7	88 ± 15.1
Serum uric acid (μmol/L)	329 ± 95.3	335 ± 65.6	386 ± 173.2	338 ± 87.1	340 ± 91.2
Fasting blood glucose (mmol/L)	5.3 ± 0.9	5.7 ± 1.1	5.5 ± 0.9	5.4 ± 0.9	5.6 ± 1.1
Serum cholesterol (mmol/L)	5.5 ± 0.8	6.0 ± 1.2	5.3 ± 0.7	5.6 ± 0.9	5.6 ± 1.0
Serum potassium (mmol/L)	4.1 ± 0.3	4.2 ± 0.4	4.2 ± 0.5	4.2 ± 0.4	4.1 ± 0.3
Serum sodium (mmol/L)	141 ± 2.4	140 ± 3.5	138 ± 2.2	140 ± 2.8	140 ± 0.3
Potassium supplementation (n)	1	0	2	3 (9.4%)	31 (9.8%)
Cerebrovascular disorder (n)	1	0	0	1 (3.1%)	17 (5.4%)
Diabetes (n)	1	1	1	3 (9.4%)	36 (11.4%)
Gestational hypertension (n)	3	1	2	6 (18.8%)	44 (14.0%)

Data for age, creatinine, uric acid, glucose, cholesterol, potassium and sodium is given as mean ± SD. Carriers of the ENaC variants did not differ significantly from non-carriers.

**Table 3:** Plasma renin activity and serum aldosterone concentration during postural and captopril challenge tests

	**Carriers of the ENaC variants**				**Non-carriers**	*P-values*
	βENaC i12-17CT	βENaC G589S	γENaC V546I	All		All variants versus non-carriers

						
**Postural test (n)**	15	8	7	30	268	
PRA, supine	0.7 (0.4–1.7)	0.7 (0.4–1.0)	0.6 (0.3–1.8)	0.7 (0.4–1.2)	0.8 (0.5–1.4)	0.37
PRA, upright	1.2 (0.8–2.3)	1.5 (0.7–2.9)	1.6 (0.3–2.4)	1.5 (0.7–2.4)	1.9 (1.0–3.5)	0.11
Aldosterone, supine	343 (225–398)	457 (251–592)	358 (238–622)	368 (243–482)	369 (273–474)	0.76
Aldosterone, upright	663 (353–1073)	725 (552–960)	1036 (585–1643)	761 (484–1129)	939 (584–1255)	0.21
						
**Captopril test (n)**	15	8	8	31	282	
PRA, 0 min	1.2 (0.8–2.4)	1.3 (0.5–2.9)	1.3 (0.2–3.4)	1.2 (0.6–2.6)	1.4 (0.7–2.5)	0.53
PRA, 60 min	1.7 (1.0–6.2)	3.0 (0.5–5.7)	2.3 (0.5–8.2)	1.9 (0.8–5.7)	3.6 (1.3–7.7)	0.12
Aldosterone, 0min	554 (302–820)	608 (474–806)	645 (361–1312)	599 (340–845)	618 (431–877)	0.47
Aldosterone, 60min	400 (245–513)	400 (336–472)	356 (261–803)	392 (250–513)	397 (311–539)	0.41

Data is given as median (interquartile range). PRA, plasma renin activity (μg/L/h); Aldosterone, serum aldosterone concentration (pmol/L). Reference values: renin 0.9–2.0 (supine) and 2.0–5.0 (upright), and aldosterone 85–470 (supine) and 220–1000 (upright).

**Table 4:** Urinary potassium excretion and relation to plasma renin and aldosterone levels

	**Carriers of the ENaC variants**				**Non-carriers**	*P-values*
	βENaC i12-17CT (n = 14)	βENaC G589S (n = 7)	γENaC V546I (n = 5)	All (n = 26)	(n = 236)	All variants versus non-carriers

						
dU-K (mmol)	86 (68–112)	85 (66–120)	80 (70–96)	83 (68–102)	79 (65–94)	0.23
dU-K/PRA upright	54 (31–122)	57 (38–180)	97 (43–274)	56 (35–129)	38 (23–84)	0.034
dU-K/PRA mean	67 (37–168)	77 (55–252)	107 (59–321)	74 (46–170)	51 (31–118)	0.048
dU-K/Aldo upright	0.15 (0.06–0.25)	0.10 (0.07–0.15)	0.08 (0.06–0.25)	0.10 (0.07–0.21)	0.08 (0.06–0.14)	0.108

Data is given as median (interquartile range). dU-K, daily urinary potassium excretion; PRA, plasma renin activity (μg/L/h); Aldo, serum aldosterone concentration (pmol/L); PRA mean, average of supine and upright PRA.
